# Fast 3D-HEVC Depth Map Coding Method Based on Spatio-Temporal Correlation and a Two-Stage Mode Decision Framework

**DOI:** 10.3390/s26020529

**Published:** 2026-01-13

**Authors:** Erlin Tian, Jiabao Zhang, Qiuwen Zhang

**Affiliations:** College of Computer Science and Technology, Zhengzhou University of Light Industry, Zhengzhou 450002, China; 2004022@zzuli.edu.cn (E.T.); 332305100569@zzuli.edu.cn (J.Z.)

**Keywords:** 3D-HEVC, depth map intra coding, intra mode decision, spatio-temporal correlation, complexity reduction in video coding

## Abstract

Efficient intra-mode decision for depth maps assumes a pivotal role in augmenting the overall performance of 3D-HEVC. Existing research endeavors predominantly rely on fast mode screening strategies grounded in texture characteristics or machine learning techniques. These strategies, to a certain extent, mitigate the complexity of mode search. Nevertheless, these approaches often fall short of fully leveraging the intrinsic spatio-temporal correlations within depth maps. Moreover, strategies relying on deterministic classifiers exhibit insufficient discrimination reliability in regions featuring edge mutations or intricate structures. To tackle these challenges, this paper presents a two-stage fast intra-mode decision algorithm for depth maps, integrating naive Bayes probability estimation and fuzzy support vector machine (FSVM). Initially, it confines the candidate mode space through spatio-temporal prior modeling. Subsequently, FSVM is employed to enhance the decision accuracy in regions with low confidence. This methodology constructs a joint mode decision framework spanning from probability screening to refined classification. By doing so, it significantly reduces the computational burden while preserving rate-distortion performance, thereby attaining an effective equilibrium between encoding complexity and performance. Experimental findings demonstrate that the proposed algorithm reduces the average encoding time by 52.30% with merely a 0.68% increment in BDBR. Additionally, it showcases stable universality across test sequences of diverse resolutions and scenes.

## 1. Introduction

In recent years, 3D video technology has found extensive applications across diverse fields, including film and television production, game development, and medical imaging. It has emerged as one of the pivotal directions in the development of multimedia technology. The multi-view plus depth (MVD) structure, through the simultaneous capture of texture images and their corresponding depth maps, empowers the system to generate high-quality virtual viewpoints by leveraging the depth-image-based rendering (DIBR) technology. This accomplishment enhances the three-dimensional visual immersion at an exceedingly low additional bit rate cost [1]. Notably, the quality of virtual viewpoints generated by the DIBR technology is highly contingent upon the coding efficiency of depth maps [2,3]. Consequently, attaining efficient depth map coding has become a crucial underpinning for the practical implementation of 3D video technology. It plays an irreplaceable and substantial role in optimizing the overall performance of the 3D-HEVC standard.

To facilitate the coexistence of sharp edges and extensive flat regions in depth maps, the 3D-HEVC standard has incorporated innovative prediction modes and coding tools, such as depth modeling modes (DMMs) and segmented DC coding (SDC). These additions are designed to preserve edge information in depth maps more efficiently [4,5,6]. Nevertheless, this optimization has presented a formidable challenge: during the intra-mode decision-making process for depth maps, it is necessary to recursively traverse all possible combinations of the 35 prediction modes of traditional HEVC and the newly introduced coding tools [7]. This procedure utilizes an exhaustive search approach to determine the scheme with the lowest rate-distortion cost. However, this operation results in an exponential growth in the computational complexity of the encoding process, thus imposing a significant limitation on the application of 3D-HEVC in real-time scenarios. Consequently, the effective reduction of the computational complexity of depth map encoding without sacrificing rate-distortion performance has emerged as a central issue that demands urgent attention in the field of 3D video coding.

Among the existing research findings, fast algorithms for depth maps can be categorized into the following two types. One type pertains to intra-frame mode prediction, which expedites the search process by pre-emptively reducing infrequent modes or skipping invalid ones. As reported in ref. [8], a high-probability initial candidate set is constructed based on the statistical probability of the depth map intra-mode. Subsequently, a simplified candidate set is chosen via a low-complexity intra-prediction cost in conjunction with an adaptive threshold. Ultimately, only the simplified set is employed to compute the complete rate-distortion cost for determining the optimal mode. The essence of this approach lies in leveraging the characteristics of the depth map and a lightweight screening mechanism to substantially reduce the computational burden of the 3D-HEVC depth map intra-mode decision, while incurring minimal degradation in coding performance. As reported in ref. [9], a rapid intra-frame mode prediction method grounded in machine learning was put forward. Leveraging the output results of decision trees (DT) corresponding to each mode as the foundation, the number of candidate modes was substantially decreased through the adaptive skipping of HEVC prediction modes and DMM during the mode decision process. Lin et al. [10] partitioned sensitive and insensitive regions by computing visual saliency indicators such as the PU gradient edge strength and the correlation with texture map details. They then integrated perceptual weighted cost and a dynamic threshold to streamline intra-frame modes. This approach significantly mitigated the intra-frame coding complexity of 3D-HEVC depth frames without compromising visual quality. As reported in ref. [11], a Deep Mode Prediction Convolutional Neural Network (DMP-CNN) was put forward to decide whether to incorporate the DMM mode into the candidate list and skip unnecessary mode decision computations. This approach trains a deep learning model to directly forecast the optimal depth modeling mode. It does so by extracting the spatial neighborhood features of the depth map prediction unit, the corresponding texture map features, and the encoding parameters as inputs. The second type aims at reducing computational complexity by accelerating the coding unit (CU) partitioning process. As reported in ref. [12], a fast CU partitioning algorithm based on decision trees was proposed. This study employed the J48 algorithm in WEKA 3.8 to construct three decision trees, each corresponding to a specific CU size. Subsequently, some attributes highly relevant to the CU partitioning decision were selected from all evaluated attributes through information gain. As reported in ref. [13], a CU partitioning algorithm grounded in boundary continuity was put forward. In this algorithm, the total sum of squares (TSS) of each boundary of the CU and the TSStotal are computed. When they are less than or equal to the pre-set threshold or the RDcost of the current optimal mode meets the criteria, the CU partitioning is prematurely terminated. As reported in ref. [14], the depth map blocks are categorized into uniform, simple edge, and complex edge types via a convolutional neural network. Based on the classification outcomes, the encoding process is adaptively streamlined: for smooth blocks, the search is terminated ahead of time; for simple edge blocks, only a limited number of modes are examined; and only for complex blocks is a comprehensive search carried out. This significantly reduces the encoding complexity of 3D-HEVC depth maps.

Based on the foregoing analysis, despite the fact that these methods have enhanced the encoding speed, several notable limitations still persist. Firstly, the majority of these methods independently exploit either spatial or temporal context information. As a result, they fail to comprehensively and profoundly explore the intrinsic spatio-temporal correlations within depth maps, thereby leading to inadequate prediction reliability. Secondly, machine learning-based approaches predominantly rely on deterministic classifiers. These classifiers exhibit poor robustness when differentiating ambiguous regions, such as depth edges and complex structures, and are highly susceptible to misjudgment. Thirdly, certain methods involve redundant processing or insufficient feature extraction for low-probability patterns. This makes it arduous to strike an optimal balance between encoding efficiency and performance.

The spatial distribution of depth maps exhibits strong continuity, and the optimal modes of adjacent prediction units (PUs) share a high degree of similarity. Moreover, in a stable scene, a notable correlation exists between the modes of PUs at the same position in the previous frame and those in the current frame. This characteristic offers valuable insights for related research. Building upon this, this paper presents a two-stage fast intra-mode decision algorithm for depth maps, which integrates naive bayes probability estimation and Fuzzy Support Vector Machine (FSVM). By effectively modeling spatio-temporal correlations and adaptively determining confidence levels, this algorithm addresses the conflict between the accuracy of mode selection and complexity control in existing methods. As a result, it attains a coordinated optimization of coding efficiency and rate-distortion performance.

This algorithm initially constructs a spatio-temporal prior-guided Bayesian framework. By leveraging the pattern information of adjacent and co-located PUs, it estimates the posterior probability of candidate patterns. The adaptive confidence is computed through probability entropy, enabling the rapid screening of high-probability candidate patterns. When the confidence level is low, the FSVM model is introduced to integrate multi-dimensional features, including edge strength, variance, and quantization parameter (QP), for fine-grained classification. This approach enhances the discrimination accuracy in complex regions. The two-stage joint decision-making mechanism of “coarse screening–fine classification” not only significantly reduces the computational cost of pattern search but also effectively guarantees the stability of coding performance.

The contributions of this paper can be summarized as follows:

1. This study presents a spatio-temporal prior-guided Bayesian framework. By exploring the pattern correlations of adjacent and co-located PUs, this framework estimates the posterior probability of candidate modes. This approach effectively shrinks the search space and bolsters the prediction reliability.

2. A normalized probability entropy confidence index is introduced to quantify the uncertainty inherent in Bayesian prediction. Additionally, an adaptive early termination mechanism is devised. When the confidence level is high, the mode can be directly selected, thereby reducing redundant computations.

3. In scenarios with low confidence, a pattern refinement strategy grounded in the FSVM is put forward. Through the integration of fuzzy membership and multi-dimensional discriminative features, this strategy enables robust classification in regions with deep discontinuities and complex structures, thus enhancing the discrimination accuracy.

The remainder of this paper is structured as follows. Section 2 presents the characteristics of 3D-HEVC intra-frame mode prediction. Section 3 meticulously elaborates on the fast depth map mode decision method proposed herein. Section 4 and Section 5 respectively provide the analysis of experimental results and the conclusion.

## 2. Observation and Analysis

**Figure 1:**
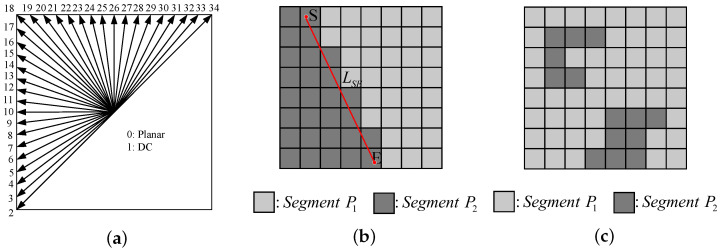
Depth intra prediction modes in 3D-HEVC: (**a**) Angular mode; (**b**) DMM1; (**c**) DMM4.

In the 3D-HEVC standard, depth map coding inherits 35 traditional intra prediction modes from HEVC [15,16], as depicted in Figure 1a. Among these, 33 intra angular modes are capable of representing diverse prediction directions. In addition to these angular prediction modes, the DC mode and Planar mode can efficiently handle smooth or approximately homogeneous regions. To retain the sharp edge characteristics of the depth map, 3D-HEVC introduces two additional depth modeling modes, namely DMM1 and DMM4 [17]. These two modes partition each prediction unit into two non-rectangular regions, with each region being characterized by a constant pixel value (CPV) [18,19,20]. For each wedge-shaped division in DMM1, as illustrated in Figure 1b, the two regions $P_{1}$ and $P_{2}$ are separated by a straight-line boundary $L_{SE}$. *S* and *E* denote the start and end points of $L_{SE}$, respectively. DMM4 can employ any contour division method, as shown in Figure 1c, and its division result is determined by the co-located reconstructed texture PU using the threshold method. Nevertheless, in the latest test model, DMM4 is no longer applicable to depth intra slices.

**Figure 2:**
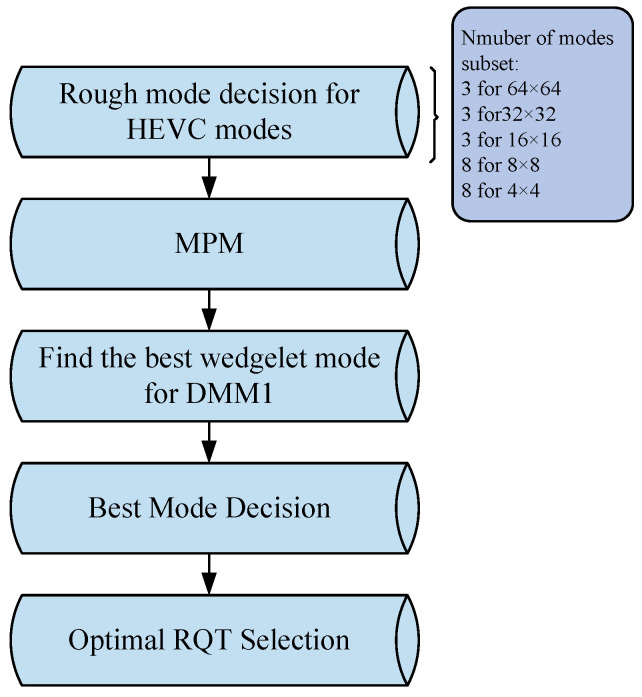
Depth intra mode decision.

As depicted in Figure 2, the flowchart of the depth intra-prediction mode decision is presented. The key process of the depth intra-mode decision can be concisely summarized into the following steps:

Step 1: The initial step in intra prediction involves conducting a rough mode decision (RMD) for the HEVC intra modes. Initially, the sum of absolute transformer differences (SATD) of the coefficients following the Hadamard transform of the residuals for 35 intra modes is computed. Subsequently, the cost is calculated using Formula (1). Finally, several candidate modes with the lowest costs are included in the list of optimal mode candidates. Specifically, for 8 × 8 or 4 × 4 size PUs, 8 modes with the minimum costs are chosen, whereas for PUs of other sizes, 3 modes with the lowest costs are selected [21].

(1) $$\mathrm{Cost}=D_{\mathrm{SATD}}+\lambda \cdot \mathrm{Rate}\left(\mathrm{Mode}\right)$$

where λ denotes the Lagrangian multiplier, and Rate(Mode) denotes the number of encoding bits required for encoding the intra-mode of the current frame.

Step 2: The optimal prediction mode of the current PU exhibits a remarkably high degree of similarity to the optimal prediction modes of the adjacent encoded PUs on its left and top. Consequently, the three optimal prediction modes of the adjacent encoded PUs on the left and top are designated as the Most Probable Modes (MPMs), denoted as MPM[0], MPM[1], and MPM[2]. Following Rate–Distortion Optimization (RDO), it is ascertained whether the current MPM is encompassed within the list of optimal mode candidates. If not, the MPM mode is incorporated into the candidate mode list and encoded as the corresponding MPM mode number [22].

Step 3: After incorporating the MPM mode into the candidate list, conduct a search for the optimally matching wedge partition among all the candidate schemes of the wedge mode and include it in the candidate mode list.

Step 4: The optimal prediction mode is derived via the RDO procedure. For each mode within the list of optimal mode candidates, the residual acquired by applying this mode undergoes transformation, quantization, and entropy encoding to yield the final coding bit rate Bmode associated with using the mode. Subsequently, the rate-distortion cost RDCost resulting from using the mode is computed through Formula (2).

(2) $$J_{\mathrm{mode}}=D_{\mathrm{SSE}}+\lambda \cdot B_{\mathrm{mode}}$$

In this context, $D_{\mathrm{SSE}}$ represents the distortion, where the sum of squared errors is employed. Specifically, it is computed as the sum of the squared differences between each pixel of the original image block and the corresponding pixel of the block reconstructed after encoding and decoding. The symbol λ denotes the Lagrange multiplier, while $B_{\mathrm{mode}}$ refers to the total number of bits required to encode this particular mode.

Step 5: The complete residual quadtree (RQT) search is carried out by utilizing the intra mode chosen in the fourth step to ascertain the optimal transform kernel size [23].

Owing to the fact that the intra-mode decision for depth map frames necessitates the examination of a substantial number of candidate modes, its encoding time constitutes approximately 86% of the total encoding time of 3D-HEVC, as reported in ref. [24]. Consequently, if the optimal prediction mode selected by the current PU can be predicted beforehand or the quantity of its candidate modes can be diminished, the encoding time can be significantly curtailed. Hence, the development of effective methodologies capable of accurately predicting the optimal intra-mode or prematurely terminating the redundant mode search is of utmost importance.

## 3. Proposed Fast Algorithm for Depth Map Mode Decision

### 3.1. Mode Probability Estimation Based on Naïve Bayes

**Figure 3:**
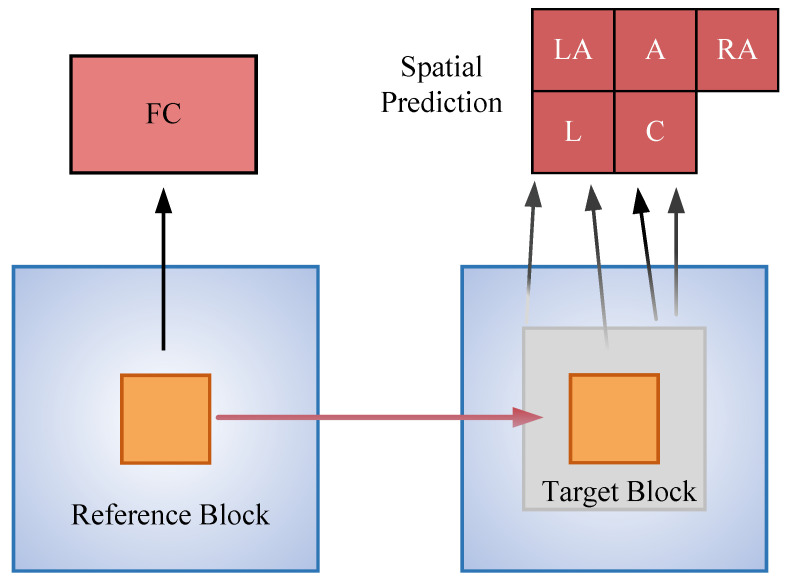
Current PU and its spatio-temporal contextual PUs. The current PU C, its spatially adjacent PUs (L, LA, A, and RA), and the co-located PU in the previous frame (FC) are shown in red. The reference block and target block illustrate the temporal correspondence between consecutive frames.

The pixel values of a depth map inherently represent the distance between objects and the camera. Their spatial distribution typically exhibits strong continuity. Simultaneously, under the premise of a stable scene structure, the CU mode at the identical position in the preceding frame demonstrates a high degree of correlation with the current PU. Specifically, this characteristic implies a high probability that adjacent PUs and the current PU are part of the same spatial area, and their optimal intra-frame modes are remarkably similar. Regarding the PU at the position corresponding to the current PU in the previous frame, when the scene does not experience substantial movement, their optimal modes generally remain consistent [25]. Based on the foregoing analysis, this paper incorporates spatio-temporal context information into the traditional Naïve Bayes model. By leveraging the prediction modes of adjacent and co-located PUs, prior constraints are imposed on the mode distribution of the current PU, thereby enhancing the accuracy of mode probability estimation. In this study, the current PU is designated as C, while the PUs to its left, upper left, above, upper right, and the co-located PU in the previous frame are denoted as L, LA, A, RA, and FC, respectively, as depicted in Figure 3. The mode information of these PUs forms the spatio-temporal context set of the current PU:

(3) Ω={L,LA,A,RA,FC}

In light of the aforementioned spatial and temporal correlations, this paper formulates a candidate mode set leveraging the neighborhood PU information. In the HEVC standard, the intra-prediction modes consist of Planar, DC, and 33 angular modes. Additionally, 3D-HEVC incorporates two depth modeling modes for depth maps. Nevertheless, in practical encoding scenarios, the optimal mode for the majority of PUs is predominantly concentrated within a limited number of common modes [26]. When probability estimation is carried out for all modes, not only does the computational burden increase substantially, but also the contribution of low-probability modes to the final decision is negligibly small, leading to only marginal improvement in overall performance. Consequently, in this paper, a candidate mode set is first generated leveraging spatial and temporal neighborhood information, and the probability is estimated only for modes with a higher likelihood. The encoded mode information of these neighboring PUs is then extracted to form the initial mode set.

(4) $$M_{\mathrm{init}}=\left\{M_{L},\;M_{\mathrm{LA}},\;M_{A},\;M_{\mathrm{RA}},\;M_{\mathrm{FC}}\right\}$$

The frequency of the occurrence of patterns within this set is meticulously counted. Patterns with higher occurrence counts are then retained as the primary candidates. Simultaneously, to bolster the stability of prediction, the Planar and DC modes are additionally incorporated. Consequently, the candidate mode set is as follows:

(5) $$M_{\mathrm{fin}}=\mathrm{TopK}\;\left(M_{\mathrm{init}}\cup \left\{\mathrm{Planar},\;\mathrm{DC}\right\}\right)$$

Here, TopK(·) selects the top-*K* modes according to empirical frequency or prior probability, where K=M denotes the total number of candidate modes. In this work, M=10, including the planar mode, the DC mode, eight angular modes, and the DMM1 mode. This candidate set covers most high-probability optimal modes, thereby preserving prediction accuracy while substantially reducing the subsequent model complexity.

The posterior probability that the current PU belongs to mode $m_{j}$ is given by the Naïve Bayes rule:

(6) $$P\left(m_{j}|\Omega \right)=\frac{P\left(m_{j}\right)\prod_{k=1}^{5}P\left(M_{k}|m_{j}\right)}{\sum_{t\in M_{\mathrm{fin}}}P\left(m_{t}\right)\prod_{k=1}^{5}P\left(M_{k}|m_{t}\right)}$$

(7) $$P\left(M_{k}|m_{j}\right)=\frac{N(M_{k},m_{j})}{\sum_{t=1}^{M}N\left(M_{k},m_{t}\right)}$$

Among these, $P(m_{j})$ denotes the prior probability of pattern $m_{j}$. $M_{k}$ represents the encoded optimal mode of the *k*-th context PU in Ω. $P(M_{k}|m_{j})$ represents the conditional probability that the adjacent or same-position CU mode is $M_{k}$ given that the current CU mode is $m_{j}$. $N(M_{k},m_{j})$ stands for the occurrence frequency where the adjacent CU mode is $M_{k}$ and the current CU mode is $m_{j}$. By means of this probability model, the posterior probability distribution corresponding to each mode in the candidate mode set can be derived, thus reflecting the likelihood of it becoming the optimal mode.

To acquire the key parameters $P(m_{j})$ and $P(M_{k}|m_{j})$, we carried out comprehensive experiments. Specifically, under the All-Intra (AI) configuration [27], four quantization parameters, namely 34, 39, 42, and 45, were set. To satisfy the demands of different resolutions, for videos in the 1024 × 768 category, Kendo and Newspaper were selected; for videos in the 1920 × 1088 category, Shark and Poznan_Street were chosen. These videos encompass a wide range of motion features and texture types. When the current PU selects the Planar mode, the probability that all five related PUs also select Planar is presented in Table 1. Adopting a similar methodology, we can derive the probability that adjacent PUs choose any mode when the current PU selects a specific intra mode. The correlation between the current PU and the PU at the same position in the previous frame is influenced by scene motion. In static or low-motion videos, this correlation is relatively strong. However, it generally remains lower than the correlation with spatially neighboring PUs.

Due to the fact that the Naive Bayes assumes feature conditional independence, the prediction results may exhibit uncertainty in certain areas (such as deep edges or high-texture regions). Consequently, this paper incorporates probability entropy to quantify the prediction confidence. Probability entropy is defined as:

(8) $$H=-\sum_{j=1}^{M}P\left(m_{j}|\Omega \right)\;\log P\left(m_{j}|\Omega \right)$$

The symbol Ω represents the set of neighboring PUs of the current PU. Specifically, Ω includes the PUs located to the left, upper-left, above, upper-right, as well as the co-located PU in the previous frame.

It is normalized to [0,1] as:

(9) $$H_{\mathrm{norm}}=\frac{H}{\log M},\;C=1-H_{\mathrm{norm}}$$

Here, *C* denotes the confidence measure and lies in the range [0,1]. A smaller entropy (i.e., a larger *C*) indicates higher model confidence. Based on this measure, an adaptive decision strategy is adopted: when *C* exceeds a threshold $C_{t}$ (i.e., the entropy is low), the prediction is regarded as stable, and the intra-prediction mode with the highest posterior probability is directly selected as the optimal mode.

(10) $$m_{\max}=\underset{j}{\mathrm{arg max}}\;P\left(m_{j}|\Omega \right)$$

When the confidence level drops below the threshold $C_{t}$, it serves as an indication that the model harbors uncertainty. At this juncture, the top three moedes exhibiting the highest probabilities are retained to constitute the candidate moede set $M_{\mathrm{cand}}$. Subsequently, the FSVM model is invoked for a refined judgment. In this study, the confidence level threshold $C_{t}$ is empirically set at 0.65. This value has been determined to strike a balance between accuracy and complexity across multiple sets of experiments.

### 3.2. Mode Decision Method Based on Fuzzy Support Vector Machine

In intra-prediction of depth map frames, the Naïve Bayes (NB) model estimates the posterior probability distribution of candidate prediction modes for the current PU by leveraging mode information from adjacent PUs and co-located PUs in the preceding frame. However, owing to the conditional independence assumption inherent in NB, the model tends to yield a more diffuse posterior probability distribution in regions characterized by complex textures, sharp depth discontinuities, or prominent edge structures. This often results in increased uncertainty or “blurring” in the predicted modes. To improve mode decision accuracy in such ambiguous regions, we integrate a Fuzzy Support Vector Machine (FSVM) with the NB probability estimation, establishing a coarse-to-fine joint decision framework. The FSVM, an extension of the conventional support vector machine, introduces a fuzzy membership degree for each training sample to represent its reliability [28]. By assigning lower membership values to noisy or boundary-ambiguous samples, their impact on the classification hyperplane is diminished during training, leading to a more stable and smoother decision boundary and thereby enhancing model robustness.

Given a training set ${\left\{\left(x_{i},y_{i}\right),s\left(x_{i}\right)\right\}}_{i=1}^{n}$, where $x_{i}$ denotes the feature vector of the *i*-th sample and satisfies $x_{i}\in R^{d}$ ($R^{d}$ is a *d*-dimension real number space), $y_{i}\in \left\{-1,+1\right\}$ is the class label, and $s(x_{i})$ is the fuzzy membership function of the sample. The membership value satisfies $0<s(x_{i})\leq 1$, and it reflects the credibility of sample $x_{i}$ belonging to its labeled class $y_{i}$.

In the framework of support vector machines, the input samples are mapped into a high-dimensional feature space through a nonlinear mapping function ϕ(·), yielding the transformed training set $\left(\phi \left(x_{i}\right),y_{i},s\left(x_{i}\right)\right)$. The separating hyperplane is expressed as

(11) $$\omega \cdot \phi (x_{i})+b=0$$

Here, ω is the weight vector and *b* is the bias term. The kernel function is defined as

(12) $$K\left(x_{i},x_{j}\right)=\phi {\left(x_{i}\right)}^{T}\phi \left(x_{j}\right).$$

Here, $\phi {\left(x_{i}\right)}^{T}$ denotes the transpose of $\phi (x_{i})$.

Generally, the formulation of the fuzzy support vector machine can be expressed as follows:

(13) $$\begin{array}{cc} \min & \left(\frac{1}{2}{∥\omega ∥}^{2}+C^{+}\sum_{i=1}^{n}s_{i}^{+}\zeta_{i}+C^{-}\sum_{i=1}^{n}s_{i}^{-}\zeta_{i}\right)\\ s.t. & y_{i}\left(\omega \cdot \phi (x_{i})+b\right)\geq 1-\zeta_{i},\;\zeta_{i}\geq 0. \end{array}$$

Here, $\zeta_{i}$ is the slack variable measuring the extent of margin violation. In Equation (13), $s\left(x_{i}\right)\in \left(0,1\right]$ denotes the fuzzy membership degree of sample $x_{i}$, which reduces the influence of noisy or unreliable samples during margin optimization. For notational convenience, we define $s_{i}^{+}=s\left(x_{i}\right)$ when $y_{i}=+1$, and $s_{i}^{-}=s\left(x_{i}\right)$ when $y_{i}=-1$. Therefore, the two membership-weighted slack penalties in Equation (13) correspond to positive-class and negative-class samples, respectively. $C^{+}$ and $C^{-}$ are the penalty coefficients controlling the contributions of the two classes.

In this paper, the FSVM refinement stage is activated only when the confidence value *C* produced by the NB model is less than or equal to the threshold $C_{t}$. Specifically, three modes $\left\{m_{a},m_{b},m_{c}\right\}$ with the highest posterior probabilities are selected from the Bayesian distribution to form the candidate mode set. Then, the posterior probabilities of the candidate modes, the normalized entropy feature, and the designed structural/statistical features are concatenated to construct the FSVM input vector:

(14) $$X=\left[P\left(m_{a}|\Omega \right),\;P\left(m_{b}|\Omega \right),\;P\left(m_{c}|\Omega \right),\;H_{\mathrm{norm}},\;T_{1},\;T_{2},\;\ldots ,\;T_{d}\right]$$

Feature extraction plays a pivotal role in learning-based mode discrimination. On one hand, the feature quality directly affects the achievable upper bound of classifier performance; on the other hand, in FSVM, the effectiveness of membership-weighting also depends on whether the features can adequately characterize the structural disparities and texture distribution of the PU. If the extracted features fail to represent the PU structure comprehensively, the classifier may still produce misjudgments even with fuzzy memberships, especially in the ambiguous regions near decision boundaries. Accordingly, this study considers both the spatial structural characteristics and statistical properties of PUs, and designs and extracts a set of highly discriminative features for FSVM training and classification, as summarized in Table 2. The definitions and roles of these features are detailed below.

$T_{\mathrm{QP}}$. The quantization parameter governs the compression intensity, with higher QP values promoting edge blurring and loss of structural detail, thereby diminishing the benefits of complex patterns and favoring simpler ones. Conversely, lower QP values preserve edge sharpness and facilitate the maintenance of optimal representation in complex patterns [29].

$T_{\mathrm{parent}\_\mathrm{mode}}$. The hierarchical prior derived from the optimal mode of the parent PU guides the candidate mode selection in the child PU, promoting consistency with the parent’s directional or modal characteristics and thus improving decision-making stability.

$T_{\mathrm{Var}}$. Variance reflects local structural complexity. Low variance typically corresponds to smooth, homogeneous regions and thus favors simpler prediction modes, whereas high variance indicates richer textures or sharper structures and often calls for more diverse directional modes to better suppress distortion.

$T_{\mathrm{edge}}$. Edge strength and orientation are closely correlated with prediction-mode direction: strong, well-defined edges typically constrain the optimal mode to a small subset of candidates aligned with the dominant edge direction, whereas weak edges provide limited directional cues and thus require a broader candidate set to mitigate misclassification.

$T_{\mathrm{mean}}$. The mean value can be used as a coarse proxy for the block-to-camera distance: regions closer to the camera typically exhibit finer structural details, which may increase the likelihood of invoking DMM evaluation.

$T_{\mathrm{firstRD}}$. The RD cost of the first mode in the RD list is considered. A small value of this parameter indicates that the coding block can be efficiently encoded via traditional HEVC intra prediction modes. In such a scenario, the evaluation process of DMM can be omitted.

Based on the foregoing analysis, we fuse these features, the posterior probabilities of the candidate patterns, and the normalized entropy into the input feature vector of the FSVM. To reduce the scale differences among heterogeneous features and improve the numerical stability of the classification process, a logarithmic compression is applied to the feature values. This operation aims to alleviate excessive variations in feature magnitudes rather than enforce a strict range constraint. Specifically, the logarithmic transformation is defined as follows:

(15) $$\hat{x}=\log_{10}\left(x\right)$$

Here, *x* denotes the raw feature and $\hat{x}$ denotes the logarithmically transformed feature.

The NB model generates different candidate mode sets $M_{\mathrm{cand}}$ for different PUs by exploiting spatial and temporal neighborhood information. Owing to the considerable heterogeneity in structural characteristics across regions, the candidate sets for each PU typically vary. Training an independent classifier for each candidate combination would greatly increase computational burden and may suffer from sparse samples, making it difficult to guarantee model stability and generalization. To address this issue, we design an FSVM scheme with global training and local decision-making: during training, a unified FSVM is learned using samples from the full mode space; during inference, the FSVM performs local discrimination only within the high-probability candidate modes provided by NB.

Let the multi-class training set be ${\left\{\left(x_{i},y_{i}\right)\right\}}_{i=1}^{N}$, where $x_{i}$ denotes the input feature vector of the *i*-th PU and $y_{i}\in \left\{m_{1},m_{2},\ldots ,m_{M}\right\}$ is the ground-truth intra-prediction mode label. Using an RBF kernel with the implicit mapping ϕ(·), the FSVM learns a discriminative function for each mode:

(16) $$f_{j}\left(x\right)=\omega_{j}^{T}\phi \left(x\right)+b_{j},\;j\in \left\{1,2,\ldots ,M\right\}.$$

where $f_{j}\left(x\right)$ denotes the decision score of the *j*th candidate mode; $\omega_{j}$ represents the weight vector of the *j*-th mode, and $b_{j}$ denotes the bias term corresponding to the *j*-th mode.

Training over the full mode space enables the FSVM to capture global decision boundaries and cross-mode dependencies, thereby improving generalization and discriminative robustness. Nevertheless, during inference, the classifier does not exhaustively evaluate all *M* modes. Instead, it performs local judgment within the candidate set $\left\{m_{a},m_{b},m_{c}\right\}$ produced by NB. The discriminative strength of each candidate mode is computed based on the input feature vector *x*, and the final predicted mode is selected as

(17) $$\hat{m}=\arg\max_{j\in \{a,b,c\}}f_{j}\left(x\right).$$

Here, $\hat{m}$ denotes the index of the mode with the highest score.

### 3.3. Overview of the Algorithm

**Figure 4:**
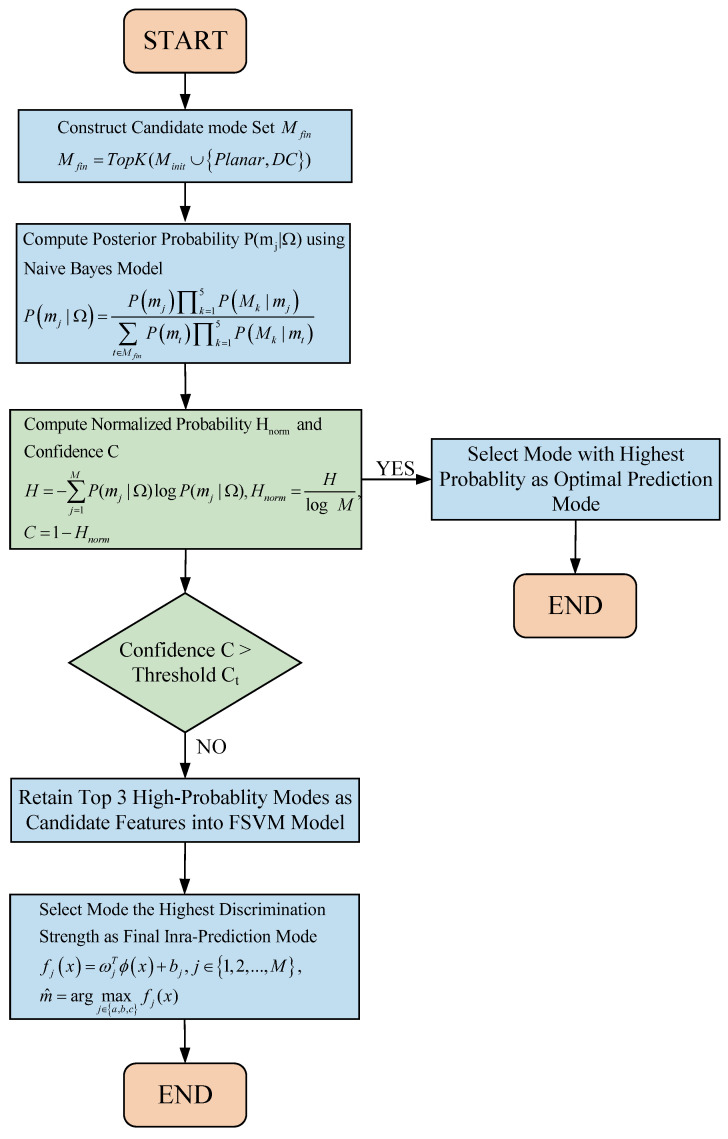
Overall algorithm flow chart.

Based on the foregoing analysis, the procedure of the algorithm proposed in this paper is as follows. Initially, the pattern information of adjacent and same-position PUs is extracted from the current PU to construct the neighborhood set Ω={L,LA,A,RA,FC}, which captures the spatio-temporal correlations. Simultaneously, a set of initial candidate patterns with high probabilities is established. Subsequently, the posterior probability $P(m_{j}|\Omega )$ of the current PU within the initial candidate pattern set is computed using the Naïve Bayes model. Moreover, the normalized probability entropy $H_{\mathrm{norm}}$ and confidence *C* are derived from the probability distribution. If the confidence exceeds the threshold $C_{t}$, it implies that the prediction result is highly accurate, and the pattern with the highest probability is directly chosen as the optimal intra-frame prediction mode. Conversely, if the confidence is lower than the threshold, it indicates that the pattern distribution is relatively scattered and the prediction result is highly uncertain. In this case, the algorithm retains the top three high-probability patterns as the candidate set and feeds their probability features along with the designed features into the FSVM model for refined discrimination. The FSVM calculates the discrimination strength of the candidate patterns based on the input feature vector and ultimately selects the pattern with the highest discrimination strength as the final intra-frame mode of the current PU. The algorithm flowchart is presented in Figure 4.

## 4. Experimental Results and Analysis

To assess the proposed 3D-HEVC depth map mode decision algorithm, we incorporated it into the 3D-HEVC reference software (HTM-16.2) [30]. The experiments were conducted on eight multiview test sequences recommended by the JCT-3V working group [31], covering two spatial resolutions, namely 1024×768 and 1920×1088. The detailed information of these test sequences is summarized in Table 3. Each sequence set consists of three texture video views and three corresponding depth map views.

All experiments were carried out strictly in accordance with the 3D-HEVC Common Test Conditions (CTC) specified by JCT-3V, under the All-Intra (AI) configuration. In this configuration, all frames are encoded independently using intra prediction only, and no inter-coded frames (i.e., P-frames or B-frames) are involved. Consequently, the effective GOP size is set to 1. The encoder was configured in a 3-view scenario, including one independent center view and two dependent side views (left and right). The encoding order followed the sequence $T_{0}$, $D_{0}$, $T_{1}$, $D_{1}$, $T_{2}$, $D_{2}$, where $T_{i}$ and $D_{i}$ denote the texture and depth frames of the *i*-th view, respectively. CTUs were fixed at 64×64 pixels with a maximum partition depth of 4, and the fast QTL option was enabled. QPs were set to {34,39,42,45} for depth-map coding and {25,30,35,40} for texture-video coding. We compare the proposed method against the 3D-HEVC encoder with full-search mode evaluation and against representative fast algorithms, as summarized in Tables 4 and 6. The coding performance is appraised in terms of encoding time and total bit rate. Specifically, BDBR represents the optimization magnitude of the total bit rate in 3D video coding, while $S_{\mathrm{time}}$ indicates the percentage variation in the overall encoding time. The calculation of $S_{\mathrm{time}}$ is presented as follows:

(18) $$S_{\mathrm{time}}=\frac{{\mathrm{Time}}_{\mathrm{proposed}}-{\mathrm{Time}}_{\mathrm{original}}}{{\mathrm{Time}}_{\mathrm{original}}}\times 100\%,$$

Among them, ${\mathrm{Time}}_{\mathrm{proposed}}$ and ${\mathrm{Time}}_{\mathrm{original}}$ denote the running times of the proposed algorithm and the original 3D-HEVC encoder, respectively.

### 4.1. A Performance Comparison and Analysis Between the Algorithm Proposed in This Paper and the Original 3D-HEVC Encoder Are Conducted

In this section, a comprehensive analysis of the simulation results regarding the algorithm’s performance will be carried out. Table 4 showcases the performance comparison data between the proposed algorithm and the original 3D-HEVC encoder. The experimental findings indicate that, while preserving comparable rate-distortion performance, the overall proposed algorithm can effectively decrease the encoding time of 3D-HEVC across all test sequences. As presented in Table 4, it is evident that for sequences with a resolution of 1024 × 768, the average encoding time is decreased by 55.37%. For sequences with a resolution of 1920 × 1088, the average encoding time is diminished by 50.45%. Among these, the Kendo sequence attains the maximum reduction of 56.47%, whereas the GT_Fly sequence reaches the minimum reduction of 47.95%. This phenomenon can be attributed to the fact that for sequences such as Kendo, the smooth regions of the depth map occupy a relatively high proportion. The Naive Bayes model can rapidly identify the high-confidence candidate set. Most prediction blocks can be directly encoded in the highest probability mode, and the number of FSVM decision-making instances is limited, thereby resulting in a more substantial reduction in encoding time. Conversely, sequences like GT_Fly feature dense edges and significant depth gradient variations. To ensure prediction accuracy, more frequent invocations of FSVM are required, which leads to a relatively smaller reduction in encoding time. Overall, the proposed algorithm is capable of attaining a dynamic equilibrium between complexity and performance in diverse scenarios, leveraging the Bayesian confidence and the FSVM decision mechanism. It reduces the encoding time by an average of 52.30% and boosts the average BDBR by 0.68%. Despite ensuring a certain level of rate-distortion performance loss, it remarkably enhances the efficiency of intra-mode decision for 3D-HEVC depth maps.

**Figure 5:**
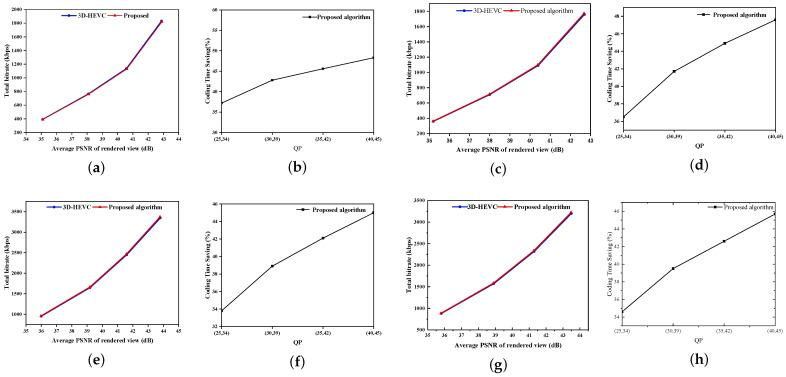
The RD performance and the reduction in encoding time of the proposed algorithm are compared with those of the 3D-HEVC encoder for four representative test sequences under different combinations of texture and depth QPs. (**a**) RD performance on the “Balloons” sequence. (**b**) Encoding-time saving curves for “Balloons”. (**c**) RD performance on the “Kendo” sequence. (**d**) Encoding-time saving curves for “Kendo”. (**e**) RD performance on the “Undo_Dancer” sequence. (**f**) Encoding-time saving curves for “Undo_Dancer”. (**g**) RD performance on the “Poznan_Hall2” sequence. (**h**) Encoding-time saving curves for “Poznan_Hall2”.

Figure 5 depicts the RD performance and the encoding time savings achieved by the proposed algorithm and the 3D-HEVC reference encoder under four representative test sequences. As shown in the figure, under various combinations of texture-video and depth-map quantization parameters, namely (25, 34), (30, 39), (35, 42), and (40, 45), the RD curves of the proposed algorithm and the baseline 3D-HEVC encoder are largely coincident. This indicates that the proposed method can maintain rate-distortion performance comparable to that of the original encoder while substantially reducing computational complexity. In terms of encoding time, the time-saving curves increase as the QP becomes larger. This phenomenon can be attributed to the following reasons. When the QP is low, due to finer PU partitioning and a more scattered mode distribution, the decision ratio of the FSVM is relatively high, resulting in limited time savings. Conversely, as the QP increases, the number of PUs decreases and the Bayesian confidence level improves. This enables the algorithm to terminate mode decisions earlier and more frequently, thereby achieving a more pronounced acceleration effect. Overall, the results demonstrate that the proposed method can strike a favorable balance between complexity and encoding performance under diverse quantization parameter conditions.

### 4.2. Ablation Experiments and Analysis

To validate the efficacy of each core module within the intra-mode decision approach for depth maps proposed in this paper, a series of ablation experiments were conducted under the HTM-16.2 reference software and the Common Test Conditions (CTC) configuration. The corresponding results are summarized in Table 5. The proposed methodology encompasses three core modules: spatio-temporal prior-guided Bayesian probability estimation, an entropy-based confidence measure for early termination, and a refined decision module based on FSVM. By selectively eliminating each module, we assessed its individual contribution to the overall coding efficiency and rate-distortion performance.

The Impact of FSVM. By comparing the “Full model” with the “model without FSVM (w/o FSVM),” it can be clearly observed that, in the absence of FSVM-based refined classification, the average BDBR increases from 0.68% to 0.89%. This indicates that FSVM provides essential and effective compensation when handling depth-discontinuous regions and complex structural patterns. Furthermore, since the fine classification step is omitted, the encoding complexity is slightly reduced, resulting in a 4.43% increase in the time-saving ratio. These findings demonstrate that the FSVM module plays a crucial role in achieving accurate mode prediction for depth blocks with complex structural characteristics.

Impact of the Confidence Mechanism. When the confidence mechanism is removed, all PUs are required to engage in the FSVM decision-making process. Consequently, the average time is significantly reduced to 41.12%. Although the BDBR of this scenario is comparable to that of the complete model, its complexity has increased substantially. This finding suggests that the confidence, serving as an effective adaptive control mechanism, can remarkably decrease unnecessary invocations of FSVM while maintaining the rate-distortion performance at a nearly unchanged level. Thus, it attains the objective of reducing the overall complexity.

Effect of Spatio-Temporal Prior. When both the spatio-temporal prior and Bayesian modeling (w/o prior) are eliminated, the algorithm performance experiences the most substantial degradation. This outcome indicates that leveraging the spatio-temporal correlation among prediction units is of utmost importance for shrinking the mode search space.

Performance of a Single-stage Classifier. The model consisting solely of FSVM is required to conduct prediction operations on 35 intra modes and DMM. Through a comprehensive comparison, it can be observed that the strategy of single-stage classification within the full mode space, relying merely on FSVM, results in an increase in BDBR to 1.04%. Meanwhile, the time savings are significantly diminished to 21.63%. These comparison results indirectly validate the significance and indispensability of the two-stage mode decision framework proposed in this paper.

Overall, the ablation experiments demonstrate that the removal of any single module results in varying degrees of rate-distortion performance degradation or an increase in computational complexity. This indicates that the full model attains the optimal balance between coding efficiency and prediction accuracy.

### 4.3. Comparative Results Between the Proposed Method and State-of-the-Art Fast Methods

To further elucidate the performance of the proposed algorithm, it is compared with representative fast methods reported in refs. [8,32], as summarized in Table 6. A fast intra-mode decision algorithm for depth map intra coding based on efficient feature selection is proposed in ref. [32]. By introducing a Self-Organizing Map (SOM) model and extracting key features such as texture information and quantization parameters, the method performs mode clustering to effectively reduce the search space of candidate modes. Consequently, the encoding complexity is significantly decreased while the quality of the synthesized view is maintained.

As shown in Table 6, the proposed method results in an average coding efficiency loss of 0.68%. In comparison, the coding efficiency losses reported in refs. [8,32] are 0.78% and 0.26%, respectively. Compared with the method in ref. [8], the proposed method reduces the coding efficiency loss by 0.10%, whereas it incurs an additional loss of 0.42% when compared with the method in refs. [32]. In terms of coding time, the proposed algorithm achieves an average time saving of 52.30%, while the methods in refs. [8,32] report average time savings of 51.5% and 39.8%, respectively. Therefore, the proposed method exhibits clear advantages in encoding speed, achieving relative improvements of 0.8% and 12.5% over the methods in refs. [8,32], respectively. Overall, the experimental results demonstrate that the proposed algorithm attains a favorable trade-off between coding efficiency and coding time.

Table 7 summarizes the comparative results between the proposed method and several recent related approaches. A hierarchical CU partition prediction model, termed Swin-HierNet, was proposed in ref. [33], which leverages the Swin Transformer architecture and multi-branch networks. By incorporating multi-branch feature fusion and a recursive decision strategy, the model enables efficient CU partitioning in the intra-frame coding of 3D-HEVC depth maps. Compared with the method in ref. [33], the proposed approach incurs a slightly higher coding quality loss of 0.33%, while achieving a 3.87% improvement in coding time saving, indicating a further reduction in computational complexity with only marginal impact on rate–distortion performance.

A convolutional neural network incorporating an attention mechanism, feature distillation, and edge perception has been proposed to predict CU partition structures in depth maps by jointly exploiting global, local, and edge-related features [34]. Compared with this method, the proposed approach achieves a higher coding time saving of 6.39%, while also reducing the coding quality loss by 0.41%.

It is worth noting that some existing approaches may achieve superior performance in certain individual metrics, such as coding efficiency or rate–distortion optimization, typically at the cost of increased model complexity or additional computational overhead. In contrast, the proposed method is designed to achieve a balanced trade-off between coding efficiency and coding quality, providing competitive performance while maintaining relatively low computational complexity.

## 5. Conclusions

This study focuses on the substantial computational complexity associated with intra-mode decision in depth maps. It presents a two-stage fast intra-mode decision algorithm for depth maps, which integrates naive Bayes probability estimation with FSVM decision-making. This approach effectively harnesses the structural correlations within both spatial and temporal neighborhoods. Initially, it accomplishes preliminary screening through candidate mode constraints and confidence determination. Subsequently, FSVM is employed to enhance the decision accuracy in complex regions. Experimental findings indicate that this algorithm significantly alleviates the overall encoding complexity while essentially maintaining the rate-distortion performance. Specifically, it achieves an average reduction in encoding time of 52.30% and only a 0.68% increase in BDBR. Moreover, it exhibits excellent stability and universality across various types of sequences.

Although the proposed two-stage fast mode-decision framework achieves a favorable trade-off between encoding complexity and Rate–Distortion performance, several limitations remain. First, the method leverages spatio-temporal priors from neighboring PUs and the co-located PU in the previous frame to narrow the candidate-mode set. In sequences with rapid motion or severe depth-map noise, the correlation of the co-located PU may weaken, reducing prior reliability and potentially biasing candidate screening. Second, the framework relies on parameters such as the confidence threshold and the number of retained candidate modes. With fixed settings, it may be difficult to maintain an optimal balance across different sequences, resolutions, and QP conditions. Future work will address these issues in two directions: (i) incorporating prior-reliability assessment with adaptive weighting to relax the candidate set or switch to a more robust candidate-generation strategy when the prior is unreliable; and (ii) developing content-adaptive schemes for thresholding and candidate selection based on QP, resolution, and scene complexity to improve cross-sequence generalization.

## Figures and Tables

**Table 1:** Probabilities of contextual PUs.

Sequence	L (%)	A (%)	LA (%)	RA (%)	FC (%)
Kendo	75.92	73.18	82.41	70.77	61.84
Newspaper	69.88	68.04	75.72	66.11	56.43
Shark	72.11	70.39	78.96	68.44	58.27
Poznan_Street	66.73	64.22	72.19	62.03	51.58
Average	71.16	68.96	77.32	66.84	57.03

**Table 2:** Summary of feature definitions and their roles.

Feature Symbol	Feature Name	Definition	Primary Role
** $T_{\mathrm{QP}}$ **	Quantization parameter	The QP of the current PU	High QP favors simple modes; low QP may benefit complex modes.
** $T_{\mathrm{parent}\_\mathrm{mode}}$ **	Optimal mode of the parent PU	The optimal intra-prediction mode selected for the parent PU	Promotes mode candidates consistent with the parent PU.
** $T_{\mathrm{var}}$ **	Variance	Pixel-value variance within the PU	Low variance → smooth; high variance → complex.
** $T_{\mathrm{edge}}$ **	Edge strength and orientation	Local edge magnitude and its dominant direction	Strong edges narrow candidates; weak edges require a wider search.
** $T_{\mathrm{mean}}$ **	Mean	Mean pixel value within the PU	Coarsely reflects detail level, affecting DMM invocation.
** $T_{\mathrm{firstRD}}$ **	RD cost of the first-ranked mode	RD cost of the top-ranked mode in the RD list	Low top-mode RD cost allows reducing/skipping DMM evaluation.

**Table 3:** Test sequences and associated parameter information.

Sequence	Resolution	Frame Rate	Frames	Three-View Input
Kendo	1024 × 768	30	200	1–3–5
Balloons	1024 × 768	30	200	1–3–5
Newspaper	1024 × 768	30	200	2–4–6
Shark	1920 × 1088	25	150	1–5–9
Undo_Dancer	1920 × 1088	25	150	1–5–9
GT_Fly	1920 × 1088	25	150	9–5–1
Poznan_Street	1920 × 1088	25	150	5–4–3
Poznan_Hall2	1920 × 1088	25	150	7–6–5

**Table 4:** Comparison of the proposed algorithm with the 3D-HEVC encoder.

Sequences	BDBR (%)	S_time_ (%)
Kendo	0.53	−56.47
Balloons	0.61	−55.38
Newspaper	0.57	−54.26
Shark	0.76	−50.32
Undo_Dancer	0.71	−49.87
GT_Fly	0.83	−47.95
Poznan_Street	0.79	−51.44
Poznan_Hall2	0.73	−52.68
1024 × 768	0.57	−55.37
1920 × 1088	0.76	−50.45
Average	0.68	−52.30

**Table 5:** Ablation study results of the proposed method.

Variant	Description	BDBR (%)	Time Saving (%)
Full	Bayesian + Confidence + FSVM	0.68	52.30
w/o FSVM	Removing FSVM	0.89	56.73
w/o Confidence	Removing the confidence mechanism	0.64	41.12
w/o Prior	Remove spatio-temporal prior (neighboring PU ignored)	1.27	33.95
FSVM-only	Global FSVM-based decision without Bayesian filtering	1.04	21.63

**Table 6:** Comparison with related works.

Sequences	This Work		[8]		[32]	
	BDBR (%)	S_time_ (%)	BDBR (%)	S_time_ (%)	BDBR (%)	S_time_ (%)
Kendo	0.53	−56.47	1.22	−50.8	0.40	−38.6
Balloons	0.61	−55.38	1.10	−50.1	0.34	−39.5
Newspaper	0.57	−54.26	1.36	−49.3	0.03	−41.8
Shark	0.76	−50.32	0.46	−51.1	–	–
Undo_Dancer	0.71	−49.87	0.25	−52.2	–	–
GT_Fly	0.83	−47.95	0.36	−51.8	0.27	−39.3
Poznan_Street	0.73	−51.44	0.54	−51.5	–	–
Poznan_Hall2	0.73	−52.68	0.96	−55.2	–	–
Average	0.68	−52.30	0.78	−51.5	0.26	−39.8

**Table 7:** Comparison of the proposed method with recent state-of-the-art methods.

Sequences	This Work		[33]		[34]	
	BDBR (%)	S_time_ (%)	BDBR (%)	S_time_ (%)	BDBR (%)	S_time_ (%)
Kendo	0.53	−56.47	0.13	−46.68	1.54	−59.53
Balloons	0.61	−55.38	0.27	−49.33	–	–
Newspaper	0.57	−54.26	0.36	−46.97	0.69	−56.41
Shark	0.76	−50.32	0.58	−52.23	–	–
Undo_Dancer	0.71	−49.87	0.21	−51.49	–	–
GT_Fly	0.83	−47.95	0.39	−48.23	1.06	−65.70
Poznan_Street	0.73	−51.44	0.41	−46.57	1.09	−53.13
Poznan_Hall2	0.73	−52.68	0.44	−45.93	–	–
Average	0.68	−52.30	0.35	−48.43	1.09	−58.69

## Data Availability

The datasets generated during and/or analyzed during the current study are available from the corresponding author on reasonable request.

## Abbreviations

3D-HEVC	3Dextensionstandard of High-Efficiency Video Coding
HEVC	High-Efficiency Video Coding
PU	Prediction Unit
RDO	Rate Distortion Optimization
QP	Quantization Parameter
BDBR	Bjøntegaard Delta Bit-Rate
CTC	Common Test Conditions
DIBR	Depth-Image-Based Rendering
HTM	3D-HEVC Test Model
FSVM	Fuzzy Support Vector Machine
NB	Naïve Bayes
RDcost	RD-Distortion cost
RDO	RD-Distortion Optimization
RMD	Rough Mode Decision
DMM	Depth Modeling Mode
